# CONSCIENTIOUSNESS, ACTIVITY ENGAGEMENT AND MOMENTARY AFFECT IN OLDEST-OLD ADULTHOOD

**DOI:** 10.1093/geroni/igz038.3289

**Published:** 2019-11-08

**Authors:** Tim D Windsor, Rachel G Curtis, Christiane A Hoppmann, Mary A Luszcz

**Affiliations:** 1 Flinders University, Adelaide, South Australia, Australia; 2 University of South Australia, Adelaide, South Australia, Australia; 3 University of British Columbia, Vancouver, British Columbia, Canada

## Abstract

Participation in meaningful activities may be particularly important for late life well-being. We examined associations of moment to moment variability in meaningful activity engagement with positive and negative affect in the daily lives of oldest-old adults. Moderating effects of conscientiousness on meaning-affect associations were also examined considering recent theorising that late life declines in conscientiousness could reflect adaptive self-regulatory processes. Participants were 73 adults aged 84 and above from the Australian Longitudinal Study of Aging Daily Life Time-Sampling (ADuLTS) study, who provided self-report data on activity engagement (including ratings of meaning and degree of challenge associated with activities) and affect on five occasions per day over seven days. Within-person variability in meaningful activity engagement was associated with positive and negative affect; however, these associations were conditional upon the extent to which activities were rated as challenging. Specifically, positive affect tended to be lower on occasions when activities were rated as less meaningful, but also more challenging. Similarly, negative affect was rated as lower on occasions when activities were regarded as more meaningful, and at the same time less challenging. Participants who were higher in conscientiousness reported higher overall positive affect, and associations of higher conscientiousness with lower momentary negative affect were evident on occasions when activities were rated as more challenging. Engagement in meaningful activity is associated with higher positive, and lower negative affect in late life, with these associations dependent on the extent to which activities are challenging. Findings are discussed in the context of self-regulatory perspectives on adaptation.

